# Expression of carbonic anhydrase IX suggests poor outcome in rectal cancer

**DOI:** 10.1038/sj.bjc.6604949

**Published:** 2009-02-24

**Authors:** E Korkeila, K Talvinen, P M Jaakkola, H Minn, K Syrjänen, J Sundström, S Pyrhönen

**Affiliations:** 1 Department of Oncology and Radiotherapy, Turku University Hospital, Savitehtaankatu 1, PB 52, Turku FIN-20521, Finland; 2 Department of Pathology, University of Turku, Kiinamyllynkatu 10, Turku FIN-20520, Finland; 3 Turku Center for Biotechnology, University of Turku, Tykistökatu 6, Turku FIN-20521, Finland; 4 Åbo Akademi University, Tuomiokirkontori 3, Turku FIN-20500, Finland; 5 Turku PET Centre, PO Box 52, Turku FIN-20521, Finland; 6 Department of Pathology, Turku University Hospital, Kiinamyllynkatu 10, Turku FIN-20520, Finland

**Keywords:** rectal cancer, CA IX, prognosis, predictive factor, radiotherapy, chemotherapy

## Abstract

The aim of the study is to assess the value of carbonic anhydrase isozyme IX (CA IX) expression as a predictor of disease-free survival (DFS) and disease-specific survival (DSS) in rectal cancer treated by preoperative radio- or chemoradiotherapy or surgery only. Archival tumour samples from 166 patients were analysed for CA IX expression by three different evaluations: positive/negative, proportion of positivity and staining intensity. The results of immunohistochemical analysis were confirmed by demonstrating CA IX protein in western blotting analysis. Forty-four percent of the operative samples were CA IX positive, of these 34% had weak and 66% moderate/strong staining intensity. In univariate survival analysis, intensity of CA IX expression was a predictor of DFS (*P*=0.003) and DSS (*P*=0.034), both being markedly longer in tumours with negative or weakly positive staining. In multivariate Cox model, number of metastatic lymph nodes and CA IX intensity were the only independent predictors of DFS. Carbonic anhydrase isozyme IX intensity was the only independent predictor of DSS, with HR=9.2 for dying of disease with moderate-intense CA IX expression as compared with CA IX-negative/weak cases. Negative/weak CA IX staining intensity is an independent predictor of longer DFS and DSS in rectal cancer.

Colorectal cancer (CRC) is a common malignancy in Western countries and the incidence is rising: there were nearly 372 000 new cases of CRC in Europe in 2002 (Ferlay *et al*, 2004). The most important prognostic factors of rectal cancer are the type of surgery, depth of invasion and nodal status. Other prognostic factors have been widely tested (Bendardaf *et al*, 2006, 2007) but as yet have not achieved an established role in the management of CRC.

Angiogenesis and tumour hypoxia have been widely studied during the past decades to develop better treatment modalities and prognostic factors. Angiogenesis favours tumour growth and metastasis, whereas hypoxia renders a tumour resistant to radiation and often to chemotherapy as well (Brizel *et al*, 1996). Hypoxic regions are common in various solid cancers due to their rapid growth. Tumour cells adapt to hypoxic conditions by stabilising the hypoxia-inducible transcription factor (HIF-1*α*), which leads to upregulation of several genes involved in cell proliferation and angiogenesis (Harris, 2002; Semenza, 2003). One of the upregulated genes is *CA9* (Opavsky *et al*, 1996). *CA9* encodes the carbonic anhydrase isozyme IX (CA IX) (Wykoff *et al*, 2000; Niemelä *et al*, 2007). Carbonic anhydrase isozyme IX is shown to be strongly inducible by hypoxia in tumour cells (Wykoff *et al*, 2000).

In earlier studies, the pattern of membranous CA IX expression is seen in malignant cells and only rarely in normal or benign cells (Pastorek *et al*, 1994; Kivelä *et al*, 2001). Colorectal tumours show an abnormal CA IX expression, which is especially seen in areas of high proliferation (Saarnio *et al*, 1998). More diffuse staining is seen in carcinomas than in benign lesions (Saarnio *et al*, 1998). Carbonic anhydrase isozyme IX is involved in maintaining the extracellular pH (Ivanov *et al*, 2001) by catalysing the reversible chemical reaction in which carbon dioxide is hydrated to carbonic acid and further to bicarbonate (Wykoff *et al*, 2000; Brennan *et al*, 2006). Thus, it is an important enzyme for cancer cells in hypoxic and normoxic conditions (Wykoff *et al*, 2000; Robertson *et al*, 2004) in the regulation of acid–base balance (Hilvo *et al*, 2007). Interestingly, in CRC samples studied by cDNA microarray (Talvinen *et al*, 2006), *CA9* was found to be the most upregulated gene.

This study was designed to assess the prognostic and predictive value of CA IX in rectal cancer treated by either short- or long course of radiotherapy (RT) with or without chemotherapy. Operative samples obtained from non-irradiated patients were used as controls. Carbonic anhydrase isozyme IX expression was studied in relation to histopathological features and clinical data pertinent to disease-free survival (DFS) and disease-specific survival (DSS).

## Patients and methods

### Study population

This study consists of archival operative tumour samples of 166 consecutive patients with rectal cancer, treated according to the standard protocols at Turku University Hospital. Patients in the preoperative treatment group had been operated during 2003–2008 and those in the control group between 2000 and 2008. To have a biologically and therapeutically homogenous study population, only tumours of the middle and lower rectum were included. Superficial tumours that had been treated by excision only were excluded. Standard staging included magnetic resonance imaging or computerised tomography (CT) of the rectum, CT of the abdomen and X-ray or CT of the thoracic area. Since 2005, the treatments have been planned by a multidisciplinary team. Thirty-seven patients were treated with long-course preoperative RT, generally by giving 50.4 Gy in 6 weeks, followed by surgery in about 4–7 weeks. Five of these patients were treated with 5-fluorouracil and 24 with capecitabine concomitantly with RT. Seventy-five patients were treated with short-course RT, consisting of five fractions of 5 Gy within 1 week and surgery on the following week. Post-operative adjuvant chemotherapy was considered for patients with lymph node-positive or high-risk lymph node-negative tumours. As a control group (*n*=54), we studied a series of patients who had not received any treatment before surgery. After completion of the treatment protocols, all patients were followed up at the Department of Surgery.

The key demographic and clinical characteristics of the patients in the three series are summarised in Table 1. Fifty-six percent of the patients were operated by anterior resection. The operation was macroscopically radical in 165 (99%) patients, microscopically radical in 154 (93%) of patients. Seventy-eight biopsy samples from the patients who received preoperative therapy were available for comparison with the respective operative samples.

The study protocol was approved by the joint Ethics Committee of Turku University and Turku University Hospital (permission no. Dnro 4/2007, 16.1.2007) and the National Authority for Medico-legal Affairs (permission no. 4423/32/300/02, 15.10.2002).

### Evaluation of the tumour response to RT

Tumour regression grade was analysed after long-course RT in the HE-stained sections according to the modified Dworak and Rödel scales, using three categories: poor, moderate and excellent response (Dworak *et al*, 1997; Rödel *et al*, 2005). The response was assessed as poor, if only minimal or no tumour regression was seen and there was a dominant tumour mass left (Dworak 0–1). When only a few tumour cells or tumour cell groups were left in the primary tumour, lymph nodes or perirectal fat, the response was assessed as moderate (Dworak 2). The response was defined as excellent, if there were only very few or no tumour cells left (Dworak 3–4). In case of moderate and excellent response, a total of 2–8 separate histological slides were studied to confirm the regression grade.

### Detection of CA IX expression

Carbonic anhydrase isozyme IX expression was analysed in all preoperative diagnostic biopsies (control group excluded) available for the study, as well as in all tumour samples obtained at operation. In each case, the most representative blocks were selected, cut to 5 *μ*m sections and subjected to immunohistochemical (IHC) staining with rabbit polyclonal antibody for CA IX (ab15086, Abcam, Cambridge, UK). The slides were pre-treated in microwave oven twice for 7 min in 10 mM sodium citrate buffer, pH 6. The antibody was diluted to a 1 : 8000 concentration in DAKO Antibody diluent-solution. The staining was carried out using the PowerVision+ Poly-HRP IHC-kit (Immunovision Technologies, Vision BioSystems, Norwell, MA, USA). The HE staining was performed according to the standard laboratory protocol.

### Analysis of CA IX expression

The IHC stainings of the samples were evaluated by two observers (EK and JS), blinded to clinical and radiological information. Light microscopes with × 4 and × 10 objectives (EK) and × 5 and × 10 objectives (JS) were used for evaluation. Carbonic anhydrase isozyme IX staining was graded using three approaches: (1) general grouping of the cases into positive or negative, (2) proportion of positive staining and (3) staining intensity. The slides were assessed as negative, if the proportion of positive carcinoma cells in the section was less than 10%. For positive cases, the proportion of positively stained carcinoma cells was analysed using four categories: (i) 10–25%, (ii) 26–50% and (iii) over 50%. The positive slides were further evaluated to determine the staining intensity. In grading the staining intensity, three categories were used: 0 for negative, 1 for weak and 2–3 for moderate to strong staining intensity. Renal cell carcinoma was used as a positive control for strong staining intensity and normal rectal mucosa as a negative control. In weak staining intensity, there was a faint positive staining in the cytoplasm and occasional staining in the cell membranes impossible to detect at a small magnification (objectives × 4 to × 5). The staining was assessed as moderate or strong, if the positive reaction in the cell membranes could easily be identified at a small magnification (objectives × 4 to × 5). If there were areas of a variety of staining intensities, the predominant intensity was chosen. In preoperative biopsies, only positive/negative staining and staining intensity were assessed, but not the proportion of staining, because of the small size of the samples.

### Western blotting of CA IX protein

To support the IHC reaction of CA IX in this study, a small tissue material from CRC patients (*n*=4) was further studied for CA IX protein. RNA from these tumours and corresponding normal colorectal mucosa had been previously isolated for cDNA microarray analysis, and *CA9* was shown to be the most upregulated gene (Talvinen *et al*, 2006). The protein from these samples was isolated to confirm CA IX expression in western blotting analysis. Total protein was quantified for each sample in duplicate by Bio-Rad Protein Assay Dye (Bio-Rad Laboratories, Hercules, CA, USA) and the quality was verified with Coomassie blue staining. For western blotting, equal amounts of denaturated protein samples were size fractioned using 10% SDS–PAGE and electroblotted onto nitrocellulose membrane (Whatman Protran, Perkin Elmer, Boston, MA, USA). Uniform loading and blotting was checked with Ponceau S staining. Carbonic anhydrase isozyme IX primary antibody (ab15086, Abcam) was diluted 1 : 1000 for western detection. Horseradish peroxidase conjugated anti-rabbit immunoglobulins (Dako, Glostrup, Denmark) and Pierce ECL Western Blotting Substrate (Thermo Scientific, Rockford, IL, USA) were used according to the manufacturers' instructions for visualisation of the signal.

### Statistical analysis

All statistical analyses were run using SPSS (SPSS Inc., Chicago, IL, USA) and STATA (Stata Corp., College Station, TX, USA) software packages (SPSS for Windows, version 16.0.2 and STATA/SE 10.1). Frequency tables were analysed using the *χ*^2^-test, with the likelihood ratio (LR) or Fisher's exact test for categorical variables. Differences in the means of continuous variables were analysed using Mann–Whitney's test or Kruskal–Wallis's test for two and multiple independent samples, respectively.

Inter-observer reproducibility of the CA IX assessments was tested using regular (Cohen's) *κ* and weighted *κ*. To calculate the latter, the ICC (intra-class correlation coefficient) test was used, with parallel mode and two-way random model, using consistency assumption and average measures option to interpret the ICC (95% CI). Concordance of CA IX expression between preoperative biopsies and operative samples was analysed using non-parametric paired-samples test (Wilcoxon signed ranks test or McNemar test). The inter-observer reproducibility of all CA IX assessments was almost perfect, with regular *κ* having values *κ*=0.988, *κ*=0.931 and *κ*=0.892, for CA IX+/−, staining proportion and intensity, respectively. Using weighted *κ* (ICC), even higher values were obtained: ICC=0.994, 0.985 and 0.984, respectively. This indicates that all three classifications of CA IX staining used in this study are highly reproducible.

Univariate survival analysis for DFS and DSS was based on the Kaplan–Meier method, where stratum-specific outcomes were compared using log-rank (Mantel-Cox) statistics. To adjust for covariates, Cox proportional hazards regression model was used, covariates being entered in stepwise backward manner. All statistical tests were two-sided and declared significant at *P*-value <0.05 level.

## Results

### General aspects of CA IX staining

**Figure 1:**
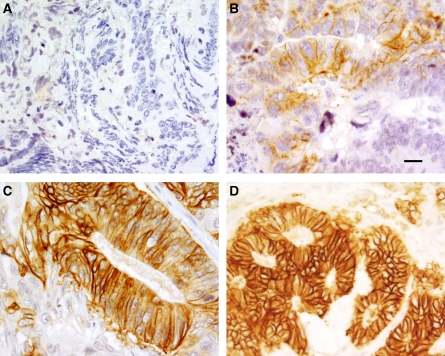
Immunohistochemistry of CA IX in rectal cancer. (**A**) Negative, (**B**) weak, (**C**) moderate and (**D**) strong staining intensity. The proportion of carcinoma cells with membranous reaction increases along with the general staining intensity (scale bar=20 *μ*m).

**Figure 2:**
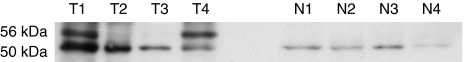
Western blotting analysis of CA IX in colorectal cancer (T) and normal colorectal mucosa (N). Strong bands with the molecular weights of 50 and 56 kDa can be seen in tumours T1–2 and T4, which were also positive for CA IX in the immunohistochemical slides. Instead, samples from the normal colorectal mucosa (N1–4) and one tumour (T3) had only a faint band of 50 kDa, all of which were also negative for CA IX in the corresponding immunohistochemical slides.

Carbonic anhydrase isozyme IX staining was positive in 49% of the diagnostic biopsies and 44% of the operative specimens. Staining intensity was weak in 15% and moderate or strong in 29% of the operative samples. The proportion of CA IX positive staining and staining intensity were directly related (Spearman *R*=0.917, *P*=0.0001). A low proportion of CA IX-positive staining (10–25%) was associated with weak staining intensity and vice versa. Negative, weak, moderate and strong staining intensity of CA IX has been illustrated in Figure 1. The results of IHC analysis were confirmed by the demonstration of CA IX protein in western blotting analysis (Figure 2).

### The expression of CA IX in the diagnostic biopsies and respective operative specimens

Biopsy samples (B) stained for CA IX were compared with the respective operative samples (S). Using CA IX positive/negative (+/−) grading, 48 out of 78 (61%) B–S pairs were concordant and 30 out of 78 (39%) were discordant. Of the discordant samples, positive CA IX expression in biopsies was downregulated in 18 out of 38 (48%) cases, and negative CA IX expression was upregulated in 12 out of 40 (30%) cases. Using CA IX intensity evaluation 43 out of 78 (55%) B–S pairs were concordant and 35 out of 78 (45%) were discordant. In pairwise comparison (Wilcoxon), CA IX expression pattern in B–S pairs was not significantly different, *P*=0.268 and 1.000 for CA IX+/− and CA IX intensity, respectively. Results of staining intensity in the biopsy specimens are shown in Table 2.

### CA IX staining intensity compared with traditional prognostic factors

In Table 3, the intensity of CA IX staining is compared with the traditional prognostic factors and survival. When the long-course RT group was divided into two treatment categories (with or without concomitant chemotherapy), there was a statistically significant difference in staining intensity (*P*=0.006) between the groups, showing more intense staining in the group that had not received concomitant chemotherapy. Tumour regression grade was possible to be evaluated in 36 long-course RT group patients. The staining was more intense in the poor response group than in the moderate/excellent response group (*P*=0.010). On the other hand, CA IX expression pattern was not significantly associated with the size, nodal status or grade of the tumour, number of examined lymph nodes, resection type or its radicality, circumferential-, proximal- or distal margins, vessel invasion, tumour necrosis or patient gender.

### Prognostic factors predicting survival

**Figure 3:**
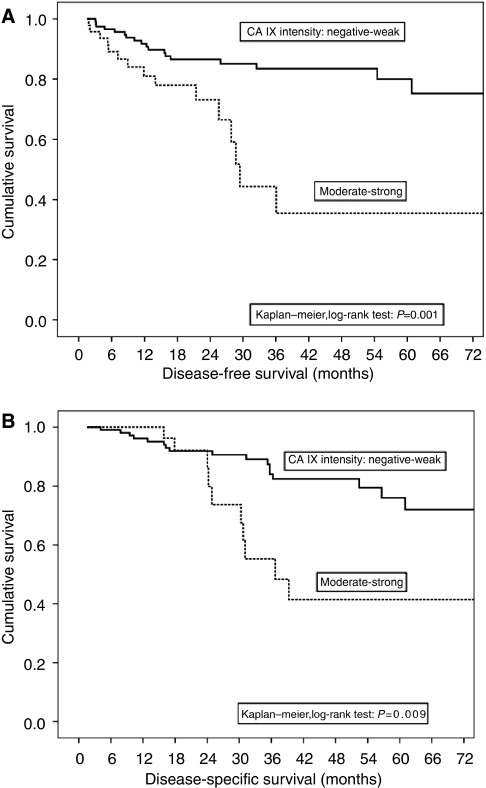
(**A**) Intensity of CA IX expression as determinant of disease-free survival in univariate (Kaplan–Meier) survival analysis. (**B**) Intensity of CA IX expression as determinant of disease-specific survival in univariate (Kaplan–Meier) survival analysis.

In univariate survival (Kaplan–Meier) analysis, patients with negative or weak CA IX staining intensity had significantly longer DFS (*P*=0.001), as depicted in Figure 3A. Also, DSS was significantly (*P*=0.009) longer among patients who had tumours with negative or weak CA IX staining intensity (Figure 3B). To analyse CA IX staining intensity as independent predictor of DFS and DSS, the following variables were entered in the multivariate (Cox) proportional hazards regression model: sex, age (young/old with median age as cutoff), treatment series (short-course RT, long-course RT and control group), preoperative tumour assessment (T), blood vessel invasion (yes/no), number of metastatic lymph nodes (LNN) (with four positive LNN as cutoff) and CA IX staining intensity. In this multivariate model, only two variables were independent predictors of DFS: (1) number of metastatic LNN; HR=4.44 (95% CI 1.37–14.38) (*P*=0.013) for disease recurrence if ⩾4 metastatic nodes and (2) CA IX intensity; HR=7.54 (95% CI 2.44–23.28) (*P*=0.003) for recurrence, if moderate-intense CA IX expression (CA IX-negative/weakly positive cases as reference) was present. In a similar Cox model, only CA IX intensity remained as significant independent predictor of DSS, with HR=9.23 (95% CI 2.26–37.64) for dying of disease with moderate-intense CA IX expression, as compared with CA IX-negative/weakly positive cases as reference. From the survival curves, the cumulative proportion of survivors at the 36-month follow-up time point was 83 and 35% for CA IX negative/weak and moderate/strong groups, respectively.

## Discussion

In this study, CA IX expression pattern was analysed from the samples of 166 patients, of whom 29 had received preoperative chemoradiotherapy, 8 patients long-course RT without chemotherapy, 75 short-course RT and 54 patients had no therapy before surgery. The intensity of CA IX expression was shown to be a significant prognostic factor in this study. When analysed with univariate (Kaplan–Meier) survival model, we found that the intensity of CA IX expression was significantly related to both DFS and DSS. Patients whose tumours were negative or weakly positive in CA IX staining had better prognosis. The difference in DFS between patients with negative/weak staining intensity and those with moderate/strong staining intensity was evident from the early postoperative period, and the difference increased over time to about 50% at 3 years. This is important while considering potential interventions with adjuvant therapies. Similar data have not been previously reported in rectal cancer.

When examined in multivariate (Cox) proportional hazards regression model controlled for several covariates, CA IX intensity together with the number of metastatic lymph nodes were shown to be independent predictors of DFS. Carbonic anhydrase isozyme IX intensity was the only significant independent predictor of DSS. These data implicate that intensity of CA IX expression seems to be a powerful independent prognostic factor, not confounded by the mode of treatment or other established prognostic factors (Giatromanolaki *et al*, 2001).

This study could not show a correlation between the expression of CA IX and other clinical or histopathological factors. Most of the patients in this study had received RT with or without chemotherapy before operation, which may have an influence on tumour size, stage and grade, nodal status and consequently, CA IX staining in the operative sample. To compensate this problem, CA IX expression was compared with the preoperative biopsies and respective surgical specimens. However, some of the available diagnostic biopsy samples were scarce, precluding adequate comparison of all sample pairs.

Carbonic anhydrase isozyme IX staining intensity in the operative samples was shown to be significantly different among the treatment categories. The same was also true among patients with different response to therapy, which was analysed from the long-course RT group specimens. Most tumours in this group that had been treated with long-course RT without chemotherapy were CA IX positive with moderate/strong staining intensity. Instead, tumours that had been treated with chemoradiotherapy were mostly CA IX negative. This may imply that chemotherapy can act as a radiosensitiser and improve tumour oxygenation and consequently also final treatment outcome. This finding can presumably have important therapeutic implications in other tumour types as well.

Tumour oxygenation is known to have an effect on patient prognosis. In advanced cancer of the uterine cervix, Hockel *et al* (1996) showed that low oxygen pressure was associated with larger tumours and more frequent parametrial spread as compared with tumours of the same stage and higher oxygen pressure. Patients with hypoxic tumours had poorer disease outcome (Hockel *et al*, 1996). Hypoxia induces CA IX, especially in or near necrotic regions (Wykoff *et al*, 2000) and in areas with high proliferation (Saarnio *et al*, 1998). Preoperative treatment can shrink the tumour and even complete remissions are seen (Ruo *et al*, 2002). As the tumour shrinks, its oxygenation may improve. Hence, preoperative therapy may reduce tumour hypoxia and thereby CA IX expression may be downregulated. In this study, there were two histologically complete responses after preoperative therapy. One complete response was unexpectedly seen after short-course RT (Marijnen *et al*, 2001), which remains to be explained but may have been related to exceptional radiosensitivity.

The present data are in alignment with the results reported in other human malignancies, where CA IX expression has been shown to be a prognostic marker as well (Table 4). This includes non-small-cell lung cancer (studied by Swinson *et al* (2003) and Giatromanolaki *et al* (2001)), bladder cancer (Hoskin *et al*, 2003), invasive breast cancer (Chia *et al*, 2001; Brennan *et al*, 2006) and oligodendroglioma (Järvelä *et al*, 2008). Interestingly, in renal cancer, low CA IX expression and absence of VHL mutation were related to a more advanced tumour and unfavourable outcome (Patard *et al*, 2008). Currently, antibodies against CA IX are being studied in phase three trials in the treatment of renal cancer (Pastorekova *et al*, 2007). Also, sulphonamides have been tested for therapeutic purposes against CA IX for several years (Pastorekova *et al*, 2007). It is possible that this strategy will also be evaluated in the treatment of other types of tumours.

To our knowledge, this is the first study to report the important prognostic significance of CA IX in rectal cancer. This study shows that strong staining intensity of CA IX is an adverse prognostic factor in rectal cancer. Further studies are needed to evaluate its potential therapeutic implications.

## Figures and Tables

**Table 1:** The clinical characteristics of the patients

	**Short-course RT, *n* (%)**	**Long-course RT, *n* (%)**	**Control, *n* (%)**	**Total, *n* (%)**	***P*-value**
*Study population*					
Female	26 (38)	13 (19)	29 (43)	68	0.069
Male	49 (50)	24 (24)	25 (25)	98	
Age (mean)	65.6	65.7	72.1	67.6	0.002
					
*Preoperative T* a					
T1–2	24 (75)	0 (0)	8 (25)	32	
T3	44 (81)	2 (4)	8 (15)	54	0.0001
T4	1 (3)	34 (92)	2 (5)	37	
TX	6 (14)	1 (2)	36 (84)	43	
					
*Postoperative T* a					
T1	3 (30)	2 (20)	5 (50)	10	
T2	29 (64)	4 (8)	15 (31)	48	
T3	40 (43)	20 (22)	32 (35)	92	0.001
T4	2 (14)	10 (71)	2 (14)	14	
No vital cancer cells left	1 (50)	1 (50)	0 (0)	2	
					
*Postoperative tumour differentiation grade (G)* b					
G1	6 (24)	7 (28)	12 (48)	25	0.043
G2	50 (47)	24 (22)	33 (31)	107	
G3	17 (59)	3 (10)	9 (31)	29	
GX	2 (40)	3 (60)	0 (0)	5	
					
*Circumferential margin (crm)* c					
0 mm	2 (18)	7 (64)	2 (18)	11	0.005
⩽1 mm	3 (33)	4 (44)	2 (22)	9	
1.1 mm⩽ crm ⩽2 mm	1 (20)	2 (40)	2 (40)	5	
>2 mm	55 (62)	17 (19)	16 (18)	88	
					
*Disease-specific outcome*					
Alive without recurrence	59 (54)	20 (18)	31 (28)	110	0.015
Alive with recurrence	5 (31)	7 (44)	4 (25)	16	
Died of disease	7 (25)	9 (8)	12 (43)	28	
Died of other causes	4 (33)	1 (8)	7 (58)	12	

RT=radiotherapy.

a T1=tumour invades submucosa, T2=tumour invades the muscular layer, T3=tumour invades through the muscular layer, T3=tumour invasion through the muscular layer, T4=tumour invasion of other organs or structures and/or perforation of the visceral peritoneum, TX=unknown.

b G1=welldifferentiated, G2=moderately and G3=poorly differentiated tumour, GX=unknown.

c crm=not measured from all tumours.

**Table 2:** CA IX expression in the three patient series

	**Short course RT, *n* (%)**	**Long course RT^a^**, ***n* (%)**	**Control, *n* (%)**	**Total, *n* (%)**	***P*-value**
*Preoperative biopsies*					
*Staining intensity*					
Negative	27 (49)	14 (56)	b	41 (51)	
Weak	18 (33)	5 (20)	b	23 (28)	0.478
Moderate/strong	10 (18)	6 (24)	b	16 (20)	
					
*Operative samples*					
*Positive proportion*					
<10% (negative)	45 (60)	21 (57)	26 (48)	92 (55)	0.387
10–25%	18 (24)	7 (19)	12 (22)	37 (22)	
26–50%	8 (11)	5 (14)	6 (11)	19 (11)	
>50%	4 (5 )	4 (11)	10 (19)	18 (11)	

CA IX=carbonic anhydrase isozyme IX; NA=Not available; RT=radiotherapy.

a Including patients treated with long-course RT with or without chemotherapy.

b Not available.

**Table 3:** CA IX expression in the operative samples related to key clinical variables

	**Staining intensity, *n* (%)**			
**Variable**	**Negative**	**Weak**	**Moderate/strong**	***P*-value**
*Preoperative therapy*				
Short-course RT	45 (60)	12 (16)	18 (24)	0.006
Long-course RT only	1 (12)	0 (0)	7 (87)	
Long-course RT+ chemotherapy	20 (69)	1 (3)	8 (28)	
Control	26 (48)	12 (22)	16 (30)	
				
*Postoperative T* a				
pT1	7 (70)	2 (20)	1 (10)	
pT2	31 (65)	5 (10)	12 (25)	
pT3	47 (51)	16 (17)	29 (31)	0.285
pT4	6 (43)	1 (7)	7 (50)	
No vital cancer	1 (50)	1 (50)	0 (0)	
				
*Postoperative nodal status (N)* b				
N1	28 (62)	6 (13)	11 (24)	0.277
N2	11 (44)	5 (20)	9 (36)	
N0	52 (55)	12 (13)	29 (31)	
NX	1 (33)	1 (33)	1 (33)	
				
*Postoperative tumour differentiation grade (G)* c				
G1	18 (72)	5 (20)	2 (8)	0.168
G2	55 (51)	16 (15)	36 (34)	
G3	16 (55)	3 (10)	10 (34)	
GX	3 (60)	1 (20)	1 (20)	
				
*Post-RT tumour regression* d				
Poor	7 (37)	1 (5)	11 (58)	0.010
Moderate/excellent	14 (82)	0 (0)	3 (18)	
				

CA IX=carbonic anhydrase isozyme IX; RT=radiotherapy.

a T1=tumour invades submucosa, T2=tumour invades the muscular layer, T3=tumour invades through the muscular layer, T4=tumour invasion of other organs or structures and/or perforation of the visceral peritoneum, TX=unknown.

b N1=1–3, N2=more than four metastatic lymph nodes, N0=no metastatic lymph nodes, NX=unknown.

c G1=well-differentiated, G2=moderately and G3=poorly differentiated tumour, GX=unknown.

d Evaluated from 36 long-course RT group patients.

**Table 4:** Carbonic anhydrase IX (CA IX) as a prognostic marker in various types of cancer

**Type of cancer**	**Prognostic/predictive significance of high CA IX Expression**	**Reference**
Non-small-cell lung cancer	Unfavourable	Giatromanolaki *et al*, 2001; Swinson *et al*, 2003; Kim *et al*, 2005; Kon-no *et al*, 2006; Simi *et al*, 2006
Bladder cancer		Hoskin *et al*, 2003
Breast cancer	Unfavourable	Chia *et al*, 2001; Brennan *et al*, 2006; Generali *et al*, 2006; Hussain *et al*, 2007; Trastour *et al*, 2007
Oligodendroglial brain tumour	Unfavourable	Järvelä *et al*, 2008
Head and neck cancer	Unfavourable	Bache *et al*, 2006; Kappler *et al*, 2008
Renal clear cell carcinoma	Favourable	Bui *et al*, 2003, 2004; Atkins *et al*, 2005; Patard *et al*, 2008
